# Climate change adaptation and local government: Institutional complexities surrounding Cape Town’s Day Zero

**DOI:** 10.4102/jamba.v11i3.717

**Published:** 2019-06-26

**Authors:** Godwell Nhamo, Adelaide O. Agyepong

**Affiliations:** 1 Institute for Corporate Citizenship, University of South Africa, Johannesburg, South Africa

**Keywords:** Climate Change, Day Zero, Water, Municipalities, SDGs

## Abstract

The challenges associated with climate change in local governments are growing daily. One such challenge is water security, an aspect that draws us to the subject matter of climate change adaptation. This article discusses findings about institutional complexities surrounding Day Zero, a concept associated with water taps running dry because of drought conditions as aggravated by climate change in the city of Cape Town, South Africa. The thrust on institutional complexities is deliberate, as this affects how crisis situations like Day Zero were handled. The data were generated mainly from the actor–actant–network theory, events study as well as document and discourse analysis methods. The actor–actant–network theory is used widely to trace how actors (humans) and actants (non-human phenomena) interact in space and time through their networks, following narratives like Day Zero, and act on climate-related matters. The analysis applied elements of grounded theory, resulting in categories and themes emerging for discussion. The article found that narratives surrounding Day Zero were embedded in both political and administrative dilemmas and red tape. Despite these challenges, the article concludes that Day Zero remains one of the landmark learning points for climate change adaptation and water security in Cape Town, South Africa, and in other cities across the world. The article recommends that Day Zero experiences continue to be embraced positively and documented further to enhance local government climate adaptation for water security currently and into the future as well.

## Introduction and background

Urban settlements, particularly metropolitan municipalities, are important platforms in addressing the negative impacts of climate change, especially adaptation. Climate change is a global phenomenon that affects all. Two main approaches for dealing with climate change are through mitigation (the act of decreasing harmful carbon emissions) and adaptation (learning to adjust and live side by side with the changing climate). The focus of this work is on adaptation. Arcanjo (2018) maintains that cities across the world suffer from water insecurity because of climate change. The changing climate results in increased average temperatures and prolonged dry weather and periods. As such, water demand under these conditions goes up with delayed or non-existent seasonal rainfall, impacting supply negatively. Drought phenomena are a common occurrence in South Africa.

When dealing with climate change, one area that remains contested is how it is now addressed as one of the common disasters under disaster risk reduction at the national level in South Africa. This orientation further speaks to other institutions that deal with disasters at the national, provincial and local government levels. *The South African Disaster Management Act of 2002* (as amended in 2015) enshrines climate change as one of the disasters to be addressed. Among some of the institutions outlined in the *Amended Act* are disaster management centres, National Disaster Management Advisory Forum, South African National Platform for Disaster Risk Reduction, South African National Defence Force (SANDF), South African Police Service and any other organs of state that may assist the disaster management structures (Republic of South Africa 2015). The act also provides for the concurrence on the declarations of disasters and expenditure on response and recovery. Reference is made to the *Intergovernmental Relations Framework Act of 2005* and the need to strengthen the representation of traditional leaders in municipal disaster management advisory forums. Of critical interest to this work is the clear reference of the *Amended Act* ‘to provide measures to reduce the risk of disaster through adaptation to climate change and developing of early warning mechanisms’ (Republic of South Africa 2015:1).

As this article was being finalised in June 2018, the South African government, through the national Department of Environmental Affairs (DEA), had published for public comments the Climate Change Bill. The objective of the bill was to build South Africa’s effective climate change response in the context of environmentally sustainable development (DEA 2018). Chapter 2 of the bill deals with policy alignment and institutional arrangements, Chapter 3 focuses on climate change response in provinces and municipalities and Chapter 5 is dedicated to national adaptation to impacts of climate change. Cross reference is made to the *Disaster Management Amendment Act of 2015*. Under Section 9, a Provincial Committee on Climate Change is established and is made up of all relevant departments and all mayors in municipalities in that province. Regarding climate change response for local government, the mayor should embark on drawing up a climate change needs assessment and response within a year of the promulgation of the *Climate Change Act* [Section 9(1)]. The needs assessment and response will be revised at least once every five years. The mayor is further required to develop and implement a climate change response implementation plan two years after the operationalising of the *Climate Change Act*. The contents of the plan are stipulated in Section 9(2), which include coming up with adaptation interventions. Working together with the national minister and the provincial leadership, the mayor will be responsible for setting up national adaptation objectives [Section 10(1)(a)]. Needless to indicate that several metropolitan municipalities in the country already have climate change response strategies and action plans and should be able to address some of the proposed new requirements.

PricewaterhouseCoopers (PwC) noted that the City of Cape Town’s population had grown significantly by about 67%, from 2.4 million people in 1996 to 4 million in 2017 (PwC). This was against a background where dam storage capability rose by a mere 15% during the same period, exhibiting a potential big crisis in the water supply. The recorded increase in population aligns with recent observations by the C40 initiative that maintains that more than 50% of the global population lived in urban areas in 2016, and this number is expected to increase to more than 70% by 2050 (C40 2018). All this, coupled with the climate change challenge, brings about pressure on water resources in cities like Cape Town. In fact, PwC recorded that the City of Cape Town was experiencing the worst drought in living memory, with dam water levels at their lowest in 19 years as of December 2017 (PwC 2017). The 2015 drought is chronicled as one in a 100-year-cycle phenomenon (Arcanjo 2018). In reference to the unfolding situation in Cape Town in August 2017, the Executive Mayor, Patricia De Lille (in Evans 2007), said:

The city had accepted it was no longer feasible to just wait for the rain. It had to start preparing for the ‘new normal’ of regular water shortages because of climate change and to stop relying solely on surface water. (n.p.)

Given the above discussion, this article focuses on teasing out how institutional complexities played out in response to the Day Zero phenomenon in Cape Town. Day Zero came about as a result of a challenge that Cape Town could encounter dry water taps at some point not so distant in future.

## Theoretical underpinnings

The Organisation for Economic Cooperation and Development (OECD) indicates that mitigation and adaptation to climate change by both public and private sectors, including local government, rest on three pillars (OECD 2007). These pillars are the development of relevant policies, investments in infrastructure and technologies and behavioural change. Mcilveen (2010) describes climate change as climatic variations lasting more than a few years. The Intergovernmental Panel on Climate Change (IPCC) defines climate change as ‘any change in climate over time, whether due to natural variability or as a result of human activity’ (IPCC 2007:21). Because the focus of this article is on climate adaptation, it is inevitable that this should be considered briefly. Climate change adaptation is defined in the IPCC Third Assessment Report (IPCC 2001:653) as the ‘adjustment in natural or human systems in response to actual or expected climatic stimuli or their effects, which moderates harm or exploits beneficial opportunities’. This definition states that both natural and human system should be able to respond to the effects of climate change or its anticipated effects. Such a response is posited to have the potential to reduce any harm that climate change may cause and at the same time take advantage of any opportunities that arise from a change in climate. The European Union (2013) indicates that climate change impacts can be predicted, and in so doing, the necessary preventive measures can be taken to minimise its effects and at the same time taking any advantage that could come out of the prediction. For cities like Cape Town, residents expect that a proper understanding in terms of water quantity and quality as well as demand and supply factors should be there to avoid insecurity. The residents further anticipate that critical, reliable and accurate information and data on climate change should be generated. The UNFCCC (2013) portrays that because the effect of climate change cannot be avoided, policies and practices focused on combating its impacts should be developed and adopted. Climate change adaptation, therefore, seeks to take the necessary steps that enable humanity to accept the reality of climate change and to build capacity to deal, especially, with the negative impacts of climate change. This is the notion commonly associated with building climate compatible and resilient cities with infrastructure and other systems that can withstand the emerging extreme weather events like flooding, hailstorms, heat waves, droughts, frost and wild fires, among others.

The United Nations Habitat III’s New Urban Agenda mentions ‘climate change’ sixteen times (United Nations 2016). In fact, the New Urban Agenda links into key global development agendas with a bearing on climate change adaptation including 2030 Agenda for Sustainable Development with its 17 Sustainable Development Goals (SDGs), the Addis Ababa Action Agenda (commonly known as the means of implementation), Paris Agreement and the Sendai Framework for Disaster Risk Reduction 2015–2030. Given space limitations, we wish to make further reference to the fact that a number of SDGs refer directly and indirectly to climate change adaptation and water. The SDGs include the following (United Nations 2015:14): end hunger, achieve food security and improved nutrition and promote sustainable agriculture (SDG 2); ensure availability and sustainable management of water and sanitation for all (SDG 6); make cities and human settlements inclusive, safe, resilient and sustainable (SDG 11); ensure sustainable consumption and production patterns (SDG 12); take urgent action to combat climate change and its impacts (SDG 13); conserve and sustainably use the oceans, seas and marine resources for sustainable development (SDG 14); and protect, restore and promote sustainable use of terrestrial ecosystems, sustainably manage forests, combat desertification, and halt and reverse land degradation and halt biodiversity loss (SDG 15).

For this article, climate change adaptation is defined through the *2015 Disaster Management Amendment Act* (Republic of South Africa 2015:2) as the human ‘process of adjustment to actual or expected climate and its effects, to moderate harm or exploit beneficial opportunities’. A similar definition is presented in the Climate Change Bill 2018. With regard to natural systems, adaptation means ‘the process of adjustment to actual climate and its effects’. Climate change refers to (Republic of South Africa 2015):

[*A*] change in the state of the climate that can be identified by changes in the variability of its properties and that persists for an extended period, typically decades or longer. (p. 4)

The fact that cities should adapt to climate change is no longer a matter of debate. In a recent publication by Filho et al. (2018), the authors focused on strengthening climate change adaptation capacity in six African cities of Douala (Cameroon), Lagos City (Nigeria), Dar-es-Salaam (Tanzania), Accra (Ghana), Addis Ababa (Ethiopia) and Mombasa (Kenya). A number of cities included in the case study are coastal cities like Cape Town. The authors realise that the extent of climate change vulnerability at any particular place (in this case, the Cape Town) is a function of the frequency and intensity of extreme weather events like droughts and floods, the portion of exposed city residents, the level of the city’s development, its wealth and economic conditions as well as prevailing political institutions and political will to prioritise adaptation strategies.

The findings from the case studies showed that the cities tend to adopt climate policies as dropped from the national level, with a limited number of them having mechanisms at the city level (Filho et al. 2018). The situation was attributed to the manner in which international climate adaptation funding and assistance is oriented, with a country-level focus instead of focusing at the coalface at the local government level. Hence, this scenario makes it difficult to implement and sustain city-wide climate policies. The case studies showed that among the key factors to enhance climate adaptation are ‘the strengthening of institutions, well designed national and city-level planning and governance’ (Filho et al. 2018:35). The next section focuses on the methodology applied for data collection and information generation as well as the analysis.

## Methodology

**FIGURE 1:**
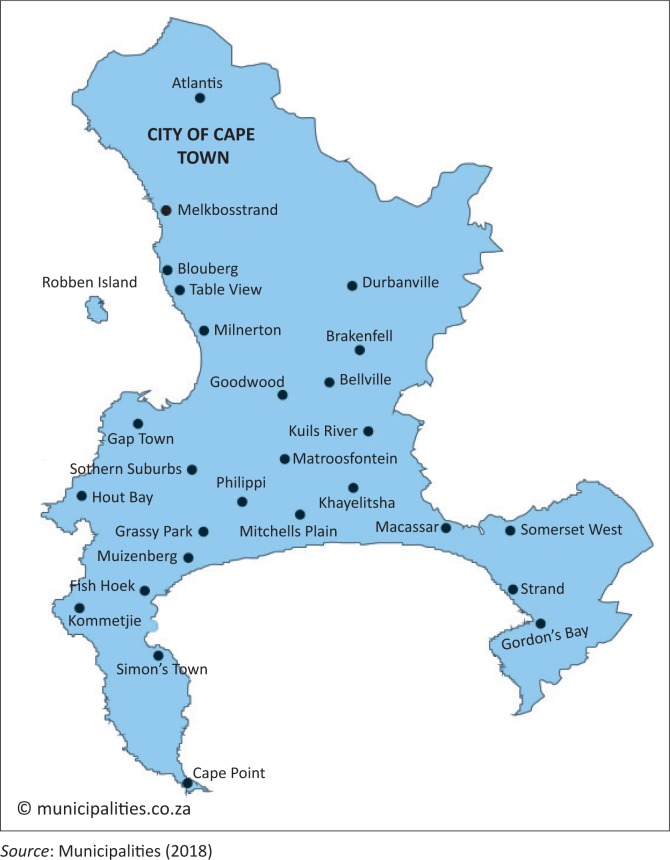
Cape Town Metropolitan Municipality.

This article set an objective to determine institutional complexities surrounding Cape Town’s Day Zero, and how this played out regarding addressing the pending water crisis resulting from prolonged droughts and the experience of a once-in-100-years drought in 2015 as discussed earlier. The Cape Town Metropolitan Municipality map is shown in Figure 1. The actor–actant–network theory (AANT) formed the broader framework of enquiry (Callon 1999). The AANT has been used successfully to trace how actors (humans) and actants (non-human phenomena) interact in space and time through their networks on climate-related matters (Nhamo 2010, 2011) or exercise their power in politics (Davies 2002). As such, AANT was deemed appropriate in establishing the nature of institutions and narratives surrounding Day Zero. Likewise, event study methods complemented AANT, as Day Zero became such a global event too loud to ignore. Although comprehensively used in the field of finance, work has started emerging using event study as a method in climate change-related work (Bimha & Nhamo 2017).

Critical document (Nhamo, Muchuru & Nhamo 2018) and discourse analysis (Nhamo & Nhamo 2017) were other supplementary methods applied in studying Day Zero. Document analysis and the analysis of words and concepts remain relevant when tracing politically charged institutions and narratives associated with Day Zero. The key documents had to be retrieved online as informed by ongoing work from the authors following the Day Zero phenomenon. In addition to critical documents, a number of articles in electronic media were retrieved. A list of critical documents retrieved, some of which are extensively analysed, is presented in Table 1.

**TABLE 1:** Key documents retrieved for Day Zero information and analysis.

Spatial scale	Key documents
**Global**	2015: Agenda for Sustainable Development with its 17 Sustainable Development Goals 2015: Addis Ababa Action Agenda 2015: Paris (Climate Change) Agreement 2015: Sendai Framework for Disaster Risk Reduction 2015–2030 2017: New Urban Agenda
**National**	2015: Disaster Management Amendment Act 2015, Act No. 16 of 2015 2018: Climate Change Bill 2018 2018: Media Release – Minister Mokonyane on Western Cape Water Crisis and Day Zero 24 January 2018
**Provincial**	2018: Premier Helen Zille’s State of the Province Address 2018 2018: Western Cape Municipal Water Security Response
**Municipal**	2017: City of Cape Town Critical Water Shortages Disaster Plan

Having collected key documents and other data, the analysis proceeded by drawing some elements from the grounded theory approach. The limited application of the grounded theory allowed the authors to determine emerging categories and, ultimately, develop key themes for further write-ups and discussions (Charmaz 2000). The next section looks at the key findings from this study.

## Analysis of data and discussion of findings

From the historical data, it was found that average dam water levels from six major dams that supply water to the City of Cape Town started deteriorating in 2015 (Fourie 2018). From a capacity of 92.5% in 2014, the dam water levels reduced drastically to 23% in 2017 (Fourie 2018) – in just a period of three years. The state of the major dams supplying water to Cape Town is shown in Table 2. In terms of drought risk, Cape Town is put under the high risk, with the other two categories being medium and low risk. In fact, the Western Cape province hosting the city had been through three consecutive droughts since 2015 (LaFrance 2018).

**TABLE 2:** Dam water levels for six largest dams supplying water to Cape Town.

Major dams	Storage		
	Capacity when full (ML)	Percentage as of 2017 – lowest since 2014	Percentage as of 22 June 2018
**Berg River**	130 010	34.1	65.9
**Steenbras Lower**	33 517	28.7	45.9
**Steenbras Upper**	31 767	58.8	88.4
**Theewaterskloof**	480 188	17.3	29.7
**Voëlvlei**	164 095	17.9	36.3
**Wemmershoek**	58 644	36.6	70.0
**Total stored (ML)**	898 221	206 926	372 483

**% storage**		23.0	41.5

*Source*: City of Cape Town (2018a)

ML, million litres.

**FIGURE 2:**
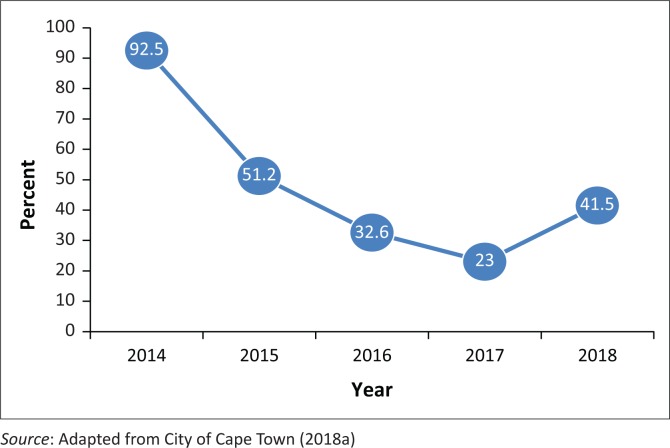
Trends in dam water levels for six largest dams supplying Cape Town (2014–June 2018).

Based on statistics from Table 2, it seems that the 2018 situation was improving compared with the whole of 2017. The trend in the six dams’ capacity since 2013 is shown in Figure 2. For planning purposes, the Western Cape Provincial Government mapped the economic impacts of the water crisis. The following were scenarios from the situation: low, uncertain, no and variable water supply (Fourie 2018) with the knock-on effect of high costs of water supply. That caused reduced productivity, increased cost of inputs, decrease in competitiveness, importation of products previously sourced locally, reputational loss as a supplier, reduced profits, job losses, delayed investments, ad hoc infrastructure investments, food insecurity, city’s reputational loss as a top tourism destination and the need for change management for the new norm. Other matters of concern were the effect of closing down businesses that led to the reduced collection of revenue from rates and taxes for the City of Cape Town. With this background, it is inevitable that readers should understand both the Day Zero concept and the institutional and political complexities associated with Day Zero. These are the sub-sections that will take up the bulk space in this analysis.

### Understanding Day Zero

Referring to Day Zero, LaFrance (2018:1) opens with a thought-provoking statement: ‘[I]t seems impossible, in South Africa where the constitution states that everyone has the right to have access to sufficient water, that the taps could be shut off’ in Cape Town. In fact, Arcanjo (2018) realised that this was the world’s first Day Zero. However, in October 2017, Cape Town developed the Critical Water Shortages Disaster Plan (City of Cape Town 2017). The Plan enshrines three phases that include water rationing (Phase I), disaster restrictions (Phase II) and full-scale disaster implementation (Phase III). The Plan was directed from the Executive Director of Safety and Security’s office and presents a number of guiding principles (see Box 1).

BOX 1 Fundamental principles for decision-making.

- Minimising the impact of the critical water shortages on human life, dignity and property
- Ensuring the continuation of critical services, such as health and safety and security services, to the public
- Ensuring the disaster is prevented from escalating by employing appropriate mitigation measures
- Ensuring the effects of the disaster on the day-to-day life of the city’s residents are reduced
- Ensuring the protection of the city’s infrastructure
- Ensuring that every person in the city has access to sufficient water to drink and is able to maintain health and hygiene
- Ensuring appropriate measures are in place to limit and respond to outbreaks of water-borne diseases and
- The most vulnerable residents of the city are least able to adapt to an escalating disaster and will require prioritised support.

*Source*: City of Cape Town (2017:6–7)

What then is Day Zero, one may continue to ask? Day Zero is not a static date as it is determined by a number of functional elements (Fourie 2018), including rainfall, temperatures and wind speed that impact evaporation rates, the availability of new supply volumes and their timing and, lastly, the consumption rate that the provincial and national governments indicated should be less than 450 megalitres (ML) a day. Day Zero was then defined ‘as the point at which the dam levels fell to 13.5%, therefore requiring all taps in the city to be shut off’ (Arcanjo 2018:2–3). Given the above discussion, Day Zero projections for Cape Town kept on migrating from 12 April through the month to 09 July, and ultimately getting lifted with an announcement on 07 March 2018 (Isaacs 2018).

**FIGURE 3:**
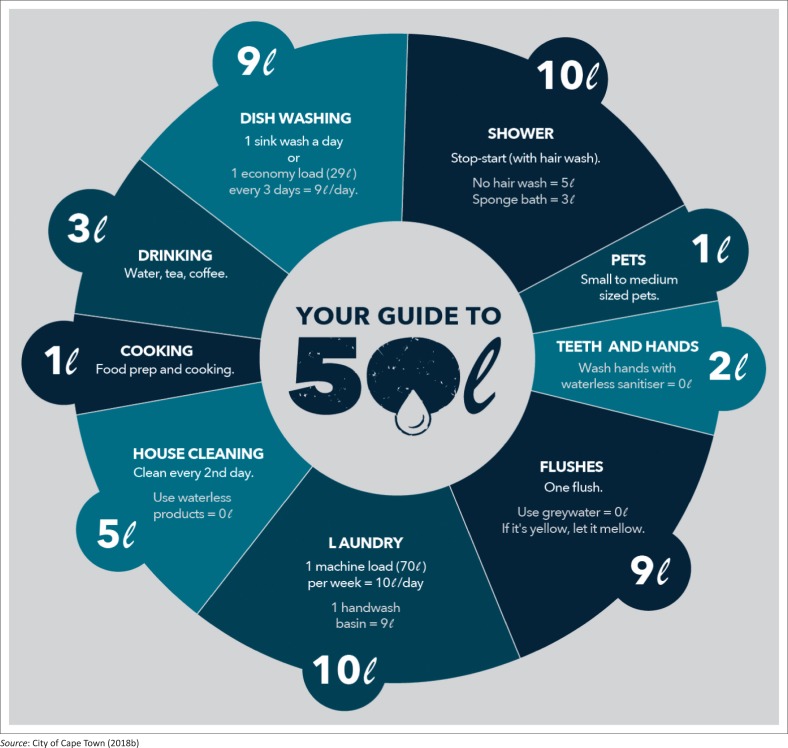
Using 50 L of water per day ‘wisely’.

Day Zero is not synonymous with zero per cent water in the dams supplying Cape Town (Jacobs 2018). This, however, comes as a wakeup call signalling an unimaginable, but real, situation that could take place if the dam storage goes to 13.5%. When this bridge is reached, it cannot be crossed as Cape Town needs to turn off most taps, apart from those supplying vital services with water. To mark the arrival of Day Zero, Capetonians (as they affectionately call themselves) had to collect water at 200 collection points planned by the political and executive heads for water distribution. An estimated 20 000 people would be in need of this water. Drawing from the World Health Organization (WHO) guidelines, each resident would be allocated a minimum of 25 L a day, with a possibility to increase it to 50 L per day. With this limited amount, the residents had to ready themselves to doing with ‘dry’ sanitation systems in their homes (Jacobs 2018). The 25 L per person per day was in sharp contrast to 60% of the residents of Cape Town who consume more than 87 L of water daily. With the proposed new daily consumption target of 450 ML per day, each resident had to be allocated 50 L per day. With this in mind, the City of Cape Town (2018b) brought up an educational poster on how to use 50 L of water allocated (Figure 3).

Although the City of Cape Town had the strategy to educate and raise greater awareness on the need to be water efficient and wise, LaFrance (2018) notes that ‘Capetonians’ did not all warm up to the campaign. As of 31 January 2018, an estimated 55% of the residents were complying with the rationed quantities. Following the declaration of Day Zero, there was great hype in the political space, complicated by the institutional set-up that has all three spheres of government with something to do regarding climate change, drought and water. The dynamics are presented in the next section.

### Institutional and political complexities surrounding Day Zero

The Cape Town metropolitan municipality is under the political leadership of the Democratic Alliance (DA). In fact, the metropolitan has been under the DA administration since 2000. However, since the August 2016 municipal elections, three more metropolitan municipalities were taken over by the DA from the national ruling party, the African National Congress (ANC), across the country. This brought about strong resentment and misgivings from the ANC. The other metropolitan municipalities that were taken over by the DA in 2016 include the city of Johannesburg and the city of Tshwane (Gauteng province) and Nelson Mandela Metropolitan (Eastern Cape province). Thinking about it, this meant that the ruling party – ANC – was no longer in charge across four major metropolitan municipalities out of the eight. In terms of significance, Cape Town hosting the National Assembly (Parliament), Nelson Mandela Metropolitan Municipality, has historical and symbolic value in terms of both carrying the world iconic leader’s name and being in his home province, the city of Tshwane is the capital city of South Africa hosting the Union Buildings and the city of Johannesburg is the country’s and Africa’s industrial hub. Such a loss to the ANC cannot be overemphasised and is likely to have bearings in the manner in which the ruling party treats Cape Town in the face of Day Zero.

South Africa has three spheres of government that include the national, provincial and local governments (Agyepong & Nhamo 2017). Apart from falling directly under the Western Cape Provincial Government that is also under the DA, Cape Town interacts with the national government under the ANC when it comes to other matters of water governance, particularly funding for bulk infrastructure, repair and maintenance. In this case, dam water levels are jointly monitored by the National Department of Water and Sanitation (DWS). This certainly brings complexities when it comes to the declaration of national disasters as these must be proclaimed from a different government, and in this case the ANC. Although the ruling political party is supposed to be separated from the governing party, in South Africa there is a very thin line between these as the ruling party has direct mandate to deploy or recall its members in government.

The Western Cape Provincial Government established the Municipal Water Security Response (Fourie 2018). This response was aimed at ensuring adequate water supply in all municipalities, including Cape Town, to avoid Day Zero. The response looked at demand-side management interventions that would reduce water consumption by every municipality. There were also supply-side interventions that were aimed at augmenting water supply to secure vital services. For Cape Town, the Provincial Government planned to pump up to 500 ML of water daily from new non-surface water until December 2021 and 200 ML per day until the end of 2018. This was to be generated from a mix of groundwater and treated wastewater. There would also be extensive communications to all stakeholders to reduce consumption to less than 450 ML a day. As of 18 June 2018, Cape Town was consuming 520 ML per day (City of Cape Town 2018b). The Provincial Government came up with key messages for its residents as presented in Box 2.

BOX 2 Key messages on the water crisis in the province.

- The effects of the current drought are of long term and can only be alleviated with 3–4 years of good rains.
- Water restrictions were there to stay until well into 2019.
- Water tariffs are likely to remain high in future.
- The Province and its cities were too dependent on rainfall and surface water sources and needed to diversify its supplies.
- Businesses need to adapt to a ‘new normal’, thus growing a business case for improved water efficiencies and own water supplies.
- Need a whole of society approach, with businesses as key partner (own operations, supply chains, employees).

*Source*: Fourie (2018:30)

The National DWS issued a directive to Cape Town, instructing it to institute a 40% water saving across its urbanised areas (City of Cape Town 2017). The directive came at the end of September 2017. The DWS indicated that Cape Town had to lower its water use to an estimated 500 ML of collective use daily. There was further anticipation that given the drought conditions in the Western Cape province, the DWS would bring more stringent restrictions on water usage.

Bornman (2018) reported that the Western Cape Premier requested the Minister of Water and Sanitation for urgent action on the water crisis in early January 2018. The premier was of the view that a joint task team should be established to work under the minister’s leadership. Such a team had to include representatives from the national, provincial and local governments. The Western Cape Government Premier was also worried that politics had taken over and pointing fingers and attributing blame to the lower tiers of government were not being helpful.

During a visit to Cape Town, the Minister of Water and Sanitation, Nomvula Mokonyane had confirmed that the bulk water supply funding was not going to be provided by the national government (Bornman 2018). This complicated issues because that mandate for bulk water supply is a DWS competency as local government was responsible for reticulation. The oversight, support, monitoring and disaster management responsibilities were allocated responsibilities and competency of the Provincial Government. The spokesperson of the DWS further shifted the responsibility to the National Department of Cooperative Governance and Traditional Affairs (COGTA), indicating that as it was a disaster, the mandate was with COGTA and money had to be allocated from COGTA’s budget. However, disaster funds from COGTA cannot be allocated just like that, as a disaster then needs to be declared. Such is the complexity of the institutional water arrangement in South Africa.

Probably the clearest institutional battles and complexities around Day Zero emerged from the media release of 24 January 2018 by the then minister of Water and Sanitation, Nomvula Mokonyane, entitled ‘Western Cape Water Crisis and Day Zero’. The minister had this to say (DWS 2018):

The Minister of Water and Sanitation, Nomvula Mokonyane, has noted several political utterances by the Premier of the Western Cape, Ms. Helen Zille, and the leader of the Democratic Alliance (DA), Mmusi Maimane, with regards to the water crisis in the Western Cape and in particular, Cape Town. What the Premier and leader of the Democratic Alliance have sought to do is to absolve themselves of their responsibilities in the management of the water crisis through an attempt to mischievously create scapegoats and shift the blame on the seriousness of the water crisis to the national government and the Minister in particular. (p. 1)

The minister went further outlining that her department had met many times with the City of Cape Town mayor and its political leadership, as well as the provincial ministry of Local Government, Environment and Development Planning with the good intention to assist in managing the drought and its negative impacts (DWS 2018). Among some of the tangible efforts was the hosting of a Water Indaba that could bring together government, the private and agricultural sectors as well as academics and experts to find solutions. One of the proposals that emerged was desalination and the minister indicated that the options had already been taken up on the ground (DWS 2018). Other options were recycling of water, increasing restrictions on the use and the clearing of canals, dredging of dams and quickening the implementation of the Berg River-Voelvlei augmentation scheme. The DWS was worried that the water use restrictions it had gazetted for the Western Cape Province were being violated, with the rate of abstraction having increased, surpassing the 45% water use restriction for domestic users and 60% for agriculture. As for Cape Town, the ‘business as usual’ approach was alleged to be as a result of lack of awareness and failure by the city to implement the restrictions. However, given the documentation on the foregone by Cape Town, one is tempted to believe that the city was doing its level best. In this regard, LaFrance wrote (2018):

As part of the Defeat Day Zero campaign, Cape Town officials have created one of the best public awareness websites I have ever seen. The Cape Town website (http://coct.co/water-dashboard/) includes the Day Zero dashboard, which provides information on alternative water supply projects, water supply levels, trends in supply level changes, and the percentage of citizens complying with the water use restrictions. There are also links that provide specific actions citizens can take to help. (p. 1)

Despite the above observations, the Minister of Water and Sanitation remained adamant, indicating that ‘[*n*]o amount of politicking and scapegoats will do away with the imminent water blackout we face in the Western Cape if we fail to act responsibly’ (DWS 2018:2). The Western Cape Premier and the DA leader were both further accused of shielding their province and political organisation from accountability. The DWS indicated that even the National Disaster Management Centre was brought into the picture by the DWS to assist. The minister then alluded to the fact that her department had successfully intervened in many other provinces that encountered similar situations in the past 3 years and would continue doing so in the Western Cape (DWS 2018):

Our mandate for water provision and support knows no politics, and we will not be drawn into petty political squabbles while the people and economy of the Western Cape are on the verge of a possible water supply blackout. (p. 2)

The minister then concluded her media statement by indicating that additional staff had been deployed in the Western Cape province to assist with compliance measures.

**FIGURE 4:**
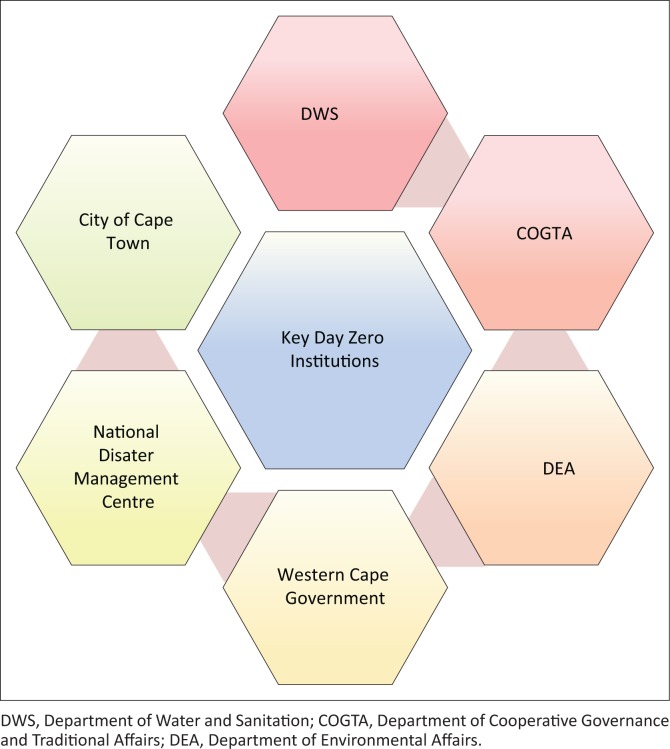
Key state institutions involved in Day Zero.

In an apparent direct response to the claims that the Western Cape Government and Cape Town were not doing enough to check compliance with water use restrictions, the Western Cape Province Premier, Helen Zille, addressed Day Zero in her State of the Province Address of February 2018. The Premier indicated that ‘Day Zero would have arrived already had restrictions not been imposed, and had Cape Town residents not put in a major water savings effort’ (Western Cape Provincial Government 2018:10). This position was further echoed by one hotel chain, which cited Jeremy Clayton of Turnkey Hospitality highlighting that Day Zero ‘escalated the need for everyone to become more environmentally conscious and the President Hotel will continue to reduce its current water consumption’ (President Hotel 2018:1). Among the interventions to be continued into the future were exploring new water sources and educating staff and guests. This would assist in reducing the group’s carbon footprint. From the Premier’s State of the Province Address, she then concluded that the province and its municipalities were not yet out of the woods if rainfall remains low in the winter of 2018 as the drought condition could be worse in the summer of 2018–2019. With the above discussion, one can present a simple mapping of the key state institutions involved in Day Zero as shown in Figure 4.

The DEA comes in significantly in Day Zero as it must perform environmental impact assessments for new water infrastructure and use of the oceans. Not shown in Figure 4, but of critical importance, are these other state institutions: National Department of Agriculture, Fisheries and Forestry, National Department of Health, Parliament, National Department of Police Services, National Department of Tourism, SANDF and other municipalities, including those as far away as the Gauteng province some 1600+ km away. Political parties (especially the ruling ANC and main opposition DA) remain key role players, with others like the Economic Freedom Fighters remaining in the picture. The ANC presents another layer of institutional complexity as it works within a tripartite composed of organised labour represented by the Congress of South African Trade Unions (Cosatu) and the South African Communist Party (SACP). This complexity is mentioned here as ministers from the tripartite (ANC, Cosatu and SACP) are those running the ministerial posts. However, in the lead to the recalling of former President Jacob Zuma, severe fault lines had developed with the SACP and Cosatu at loggerheads with the ANC. Civil society is also another key actor, with the Gift of the Givers, a global non-governmental organisation (NGO) whose hometown is Cape Town, being heavily involved in Day Zero water donations campaign from across the country.

Talking of Day Zero, the national water donation campaign programme laid bare further institutional complexities and political conflicts. Lindeque (2018) reported a warning given by the DWS through its spokesperson, Sputnik Ratau, for Gauteng province residents to ‘be careful not to create their own water crisis’ as they donated bottled water to Cape Town. In fact, Ratau was cited on 01 February 2018 highlighting that ‘[*t*]he situation in Cape Town‚ although dire‚ is not in a place really where we need to be cutting water from all over the country towards it’ (Saal 2018). The call was made against a background when the NGO Gift of the Givers had initially shipped 100 000 L of water to Cape Town on Monday 29 January 2018. The water was being delivered to the SANDF military base at Fort Ikapa. All water was to be stockpiled in SANDF military bases in Western Cape awaiting distribution to needy areas as appropriate.

As indicated earlier, on 07 March 2018, the leader of the official opposition political party, Mmusi Maimane, announced that Day Zero had been defeated (Isaacs 2018). The announcement was not received with enthusiasm from all circles as some called for the DA leadership to be criminally charged. This was the view from some activists like the Stop COCT action group who questioned how the city had such a dramatic turnaround in a relatively short period. In Stop COCT’s view, the DA leader had started electioneering for the 2019 national elections by claiming the victory over Day Zero. However, representatives of the Water Crisis Coalition saluted the ‘Capetonians’ for saving water and taking the city call seriously. This was not all good news as the Crisis Coalition simultaneously brought another institutional matter demanding for an independent investigation into tenders that were associated with the water restrictor devices. Water collection points planned for Cape Town were procured for a cost of about R200 million.

### Ethical consideration

This research used secondary data and information from publicly available online sources. The authors further adhered to conducting such research in an ethical matter related to the use of secondary data, information and sources.

## Conclusion

Like it is commonly said, necessity brings major interventions and inventions. The City of Cape Town is now at a very advanced stage in terms of addressing climate change adaptation, especially as it relates to water security. The city created a world-class platform to educate and raise awareness not only among its residents but also across South Africa and the world. New desalination plants have mushroomed along with other underground water abstraction points. Day Zero matters are now raised in global platforms including the C40 climate initiative, and this is likely to continue as more cities across the world face similar challenges. With this experience, South Africa’s political administration has learnt to work together for the welfare of the citizens regardless of where the municipalities are located (ANC or DA run). Day Zero further showed the critical role that academic and other research institutions can play. From the Cape Town experience, bottled water came from all the corners of South Africa until the DWS raised concern with shifting the problem from one place to the other. Although this could have been politically charged, it remains a fact that the act could have resulted in creating a new water challenge in Gauteng and other areas. Whether this was politics or genuine remains another research focus outside this article.
